# Disease and Economic Burden of Hospitalizations Attributable to Diabetes Mellitus and Its Complications: A Nationwide Study in Brazil

**DOI:** 10.3390/ijerph15020294

**Published:** 2018-02-08

**Authors:** Michelle Quarti Machado Rosa, Roger dos Santos Rosa, Marcelo G. Correia, Denizar V. Araujo, Luciana R. Bahia, Cristiana M. Toscano

**Affiliations:** 1 Internal Medicine Department, State University of Rio de Janeiro, Rio de Janeiro 20551-030, Brazil; denizarvianna@gmail.com (D.V.A.); lucianabahia@gmail.com (L.R.B.); 2 Social Medicine Department, School of Medicine, Federal University of Rio Grande do Sul, Porto Alegre 90035-003, Brazil; roger.srosa@gmail.com; 3 Biostatistics and Bioinformatics Department, National Institute of Cardiology, Rio de Janeiro 22240-006, Brazil; mgoulart.inc@gmail.com; 4 Collective Health Department, Federal University of Goiás, Goiânia 75345-000, Brazil; ctoscano@terra.com.br

**Keywords:** diabetes mellitus, cost and cost analysis, hospitalization, inpatients, health care expenditure, cardiovascular disease, chronic non-communicable disease

## Abstract

Diabetes is associated with a significant burden globally. The costs of diabetes-related hospitalizations are unknown in most developing countries. The aim of this study was to estimate the total number and economic burden of hospitalizations attributable to diabetes mellitus (DM) and its complications in adults from the perspective of the Brazilian Public Health System in 2014. Data sources included the National Health Survey (NHS) and National database of Hospitalizations (SIH). We considered diabetes, its microvascular (retinopathy, nephropathy, and neuropathy) and macrovascular complications (coronary heart disease, cerebrovascular disease, and peripheral arterial disease), respiratory and urinary tract infections, as well as selected cancers. Assuming that DM patients are hospitalized for these conditions more frequently that non-DM individuals, we estimated the etiological fraction of each condition related to DM, using the attributable risk methodology. We present number, average cost per case, and overall costs of hospitalizations attributable to DM in Brazil in 2014, stratified by condition, state of the country, gender and age group. In 2014, a total of 313,273 hospitalizations due to diabetes in adults were reported in Brazil (4.6% of total adult hospitalization), totaling (international dollar) Int$264.9 million. The average cost of an adult hospitalization due to diabetes was Int$845, 19% higher than hospitalization without DM. Hospitalizations due to cardiovascular diseases related to diabetes accounted for the higher proportion of costs (47.9%), followed by microvascular complications (25.4%) and DM per se (18.1%). Understanding the costs of diabetes and its major complications is crucial to raise awareness and to support the decision-making process on policy implementation, also allowing the assessment of prevention and control strategies.

## 1. Introduction

Non-communicable diseases (NCD) are the leading cause of disability and mortality globally, being responsible for 39.5 million deaths in 2015 [1]. Diabetes mellitus (DM) is one of the four major NCDs, together with cardiovascular diseases, cancer, and chronic respiratory diseases [2]. Diabetes prevalence is rising, representing a growing challenge to public health. A total of 415 million people were estimated to be diagnosed with diabetes worldwide in 2015, and it is expected that this number will rise to 642 million by 2040 [3]. One study demonstrated worldwide prevalence trends increasing from 4.3 to 9.0% in men, and from 5.0 to 7.9% in women from 1980 to 2014, with steeper increase in low and middle-income countries [4]. Brazil ranks fourth in the world in number of individuals with diabetes [4]. The 2013 Brazilian National Health Survey (NHS) demonstrated self-reported prevalence of diabetes of 6.2% in the population aged 18 years or older, reaching 19.9% in those aged 65–74 years [5]. This prevalence is certainly underestimated given other previous Brazilian studies with laboratory confirmation, which have shown that approximately half of individuals with diabetes were unaware of the diagnosis [6,7].

Studies have demonstrated that people with diabetes are at higher risk of hospitalization [8,9,10,11] and readmission than people without diabetes [12,13]. The diabetes economic burden is significant and is expected to increase over time. Global health expenditures related to diabetes and its complications were estimated at $673 billion in 2015 [3]. Such costs represent a significant portion of national health expenditures, varying from 2.5 to 15% by country, depending on availability and access to healthcare services [14].

In the early 2000s, Brazil initiated a series of strategies aiming at increasing access of the population with diabetes and hypertension to healthcare services [15] and providing early diagnosis for diabetes through a national population-based screening program [16,17]. Later, a National Strategic Plan for chronic NCD was developed and implemented [18]. This plan, in accordance to the World Bank and the International Diabetes Federation, recommends countries conduct national studies of cost of illness and economic burden of diabetes [3].

Healthcare in Brazil is provided by both public and private sectors. Public healthcare services are provided by the National Unified Health System (SUS), which offers, free of charge, universal health access covering about 75% of the population in the country [19]. 

Demographic, epidemiological and nutritional transition processes, urbanization and economic and social growth contribute to the greater risk of developing chronic NCD. Diabetes, stroke, myocardial infarction, hypertension, cancer and chronic respiratory diseases account for about 80% of deaths in Brazil, reaching heavily poor sections of the population and more vulnerable groups, such as the population with low schooling and income [18].

The full economic burden of diabetes in Brazil is still unknown. Hospitalization costs associated with diabetes and its complications are reported to be the most significant portion of direct medical costs. In this study, we estimated the number of hospitalizations due to DM and its complications and their economic burden in Brazil.

## 2. Materials and Methods 

### 2.1. Study Design, Site and Population

We estimated the number of hospitalization due to DM and its complications in 2014 in Brazil, as well as its costs. We considered hospitalizations occurring in adults aged 20 years and older in all 27 states in the country, through SUS.

### 2.2. Ethics Approval

The Ethics Committee of Federal University of Goias in Goiania, Brazil, granted ethical approval for this investigation in October 2014 (# 852808). Considering we used secondary publicly available data, with no personal identifiers, the Institutional Research Board (IRB) waived written individual consent.

### 2.3. Data Sources

#### 2.3.1. Diabetes Prevalence

The prevalence of self-reported diabetes was obtained from the 2013 NHS [5], stratified by gender, age groups and state within the country. The original NHS database was analyzed and estimates of self-reported diabetes prevalence were generated considering a positive response of surveyed individuals to question Q030 “Has any doctor ever given a diagnosis of diabetes?”, excluding individuals reporting diagnosis of gestational DM (as responded in a different question of the survey). Evidence suggests that self-report of a physician’s diagnosis of diabetes is a good estimate of diagnosed diabetes [20]. 

As individuals with diabetes who are unaware of the disease may also be hospitalized due to diabetes or its complications, we considered the prevalence of undiagnosed diabetes for this study. To account for undiagnosed diabetes, the prevalence of self-reported diabetes was multiplied by a factor of 2, based on recent evidence from the Brazilian literature, indicating that half of the individuals with diabetes diagnosis by laboratory confirmation were unaware of their disease [7]. This strategy has been applied by other authors for the estimation of diabetes disease burden [21]. 

#### 2.3.2. Hospitalization and Cost Data 

All hospitalizations occurring nationwide in SUS are recorded in a National Hospitalization Information System (SIH), which includes information on hospital admissions and discharges and its costs to the SUS. We obtained raw hospitalization data from SIH-SUS, without personal identification information, which are publicly available online [22]. 

A standardized hospital admission form (AIH) reports the main hospitalization diagnosis. We considered two types of AIH: AIH-1 (conventional hospitalization authorization) for the estimates of diabetes hospitalizations, and AIH-5 (long-term hospitalization authorization) considered in addition to AIH-1 for economic burden estimates.

International statistical classification of diseases and related health problems, 10th revision (ICD10) codes [23] assigned for admission diagnoses of all hospitalized patients are recorded in the SIH. The databases were extracted in October/2016, and the following variables were considered: type of AIH, state of residence, sex, age group, date of admission, admission diagnosis, and cost of hospitalization. The data were extracted and analyzed in Microsoft Excel^(R)^ Office Excel (R) 2007 (12.0.4518.1014) MSO (12.0.4518.1014) spreadsheets.

#### 2.3.3. Population Data 

Considering 2014 as the base year for this cost analysis, population estimates for 2014, by age group, and gender for each state were obtained from the National Institute of Geography and Statistics. The total adult population (20 years and older) in 2014 was estimated at 137,640,060 inhabitants [24]. The age groups were divided in 5-year strata from the age of 20. Standardized hospitalization rates adjusted by gender and age group were calculated using direct standardization method.

### 2.4. Outcomes of Interest

We considered hospitalizations due to diabetes and its complications. Complications included microvascular (retinopathy, nephropathy, and neuropathy), macrovascular (coronary heart disease, cerebrovascular disease, and peripheral arterial disease), respiratory and urinary tract infections, as well as selected cancers. Hospitalizations for DM in pregnancy (ICD-10 O24) were excluded.

Hospitalizations were thus categorized into two groups: (1) those in which the main diagnosis was reported as diabetes and coded as ICD10 E10 to E14; (2) those in which the main diagnosis was reported as any chronic complication of DM and related diseases, including infectious and neoplastic diseases for which DM is considered to be an important risk factor. The list of diagnosis included in this second group was adapted from the one considered by the American Diabetes Association’s study of economic costs of diabetes in the US [25], and included 66 diagnosis as coded by ICD-10 three-digit codes (Supplementary Table S1).

### 2.5. Relative Risks 

For each and all chronic complications of DM and related conditions considered, we obtained individual relative risks of hospitalization for people with diabetes compared to those without the disease. Relative risk estimates for each diagnosis were obtained through systematic literature reviews (Supplementary Table S1).

### 2.6. Data Analysis

#### 2.6.1. Burden of Hospitalizations Attributed to Diabetes and Its Complications

Except for hospitalizations in which the main diagnosis was reported as DM, the proportion of hospitalizations due to DM was estimated using the attributable risk methodology. This method considers that diabetic patients use healthcare services more than non-diabetics and that a portion of the care associated with such medical care can be attributed to diabetes. The risk of presenting a particular medical condition, according to the presence or absence of DM, and the proportion of the population with the disease are combined to calculate the etiological fraction.

The etiological fraction for each of the 66 conditions considered were calculated using the following formula:

RAPI = [P × (iRR − 1)]/[P × (iRR − 1) + 1],(1)

where RAPI is the fraction of risk attributable to the population for the medical condition “i” due to diabetes, P represents the prevalence rate of diabetes in each state by gender and age group, and iRR is the relative risk of hospitalization for people with diabetes compared to those without the disease.

A total of 702 demographic strata were generated, resulting from a combination of 2 sex categories, 13 age categories (i.e., 20–24, 25–29, 30–34, 35–39, 40–44, 45–49, 50–54, 55–59, 60–64, 65–69, 70–74, 75–79 and 80+ years), and 27 states. The number of hospitalizations for each of the conditions considered, by each of these 702 demographic strata (sex, age group, and state) was obtained.

Specific etiological fractions were then applied to the number of hospitalizations obtained for each of the 702 demographic strata, resulting in 46,332 estimates (66 ICD-10 codes multiplied by 702 demographic strata) of the proportion of hospitalizations attributable to DM. 

Results were further grouped in four age groups (20–44, 45–64, 65–74 and 75+ years old), and are reported by sex, age group and main diagnosis. Diagnosis groups considered for reporting results are DM, cardiovascular disease, kidney disease, eye disease, neurological disease, infectious disease, and neoplasms.

Proportion of hospitalization and population hospitalization rates (per 10,000 population aged 20 years and older) are reported, comparing overall hospitalizations and those due to diabetes.

#### 2.6.2. Direct Medical Costs Attributed to Diabetes and Its Complications

Economic burden analysis considered the SUS perspective as payer. A top-down costing methodology was used considering on the combination of prevalence and relative risks [26]. This methodology allocates to diabetes a portion of the total expenditures of hospitalizations (for several conditions) that could be due to the diabetes, based on the estimate of the proportion of total services consumed by individuals with the disease [27,28] as described above.

Hospitalization charges are based on Diagnostic Related Groups (DRG), with addition of values resulting from on intensive care unit (ICU) stay, certain special medications, prostheses and other selected materials. In addition to these direct medical costs, which include hospital stay, staff, diagnostic and therapeutic procedures, materials and drugs, non-medical costs for hospital stay of a parent or caregiver accompanying the hospitalized individual is also included. Reimbursed values by cost items are standardized nationwide based on SUS own price list [29].

Monetary values were obtained in Brazilian reais (R$) and then converted to international dollars (Int$) considering the purchasing power parity (PPP) (conversion factor 1.748) [30].

Total hospitalization costs and average cost per hospitalization, by diagnostic groups, and by specific hospitalization cause are presented, comparing all hospitalizations and those attributed to diabetes. Costs are further presented stratified by gender and age group. 

## 3. Results

We considered the national prevalence of undiagnosed diabetes as 12.4%, varying by age group and state (Supplementary Table S2). As such, we estimated that 17,320,339 adult individuals in the country would have diabetes. 

A total of 11.3 million hospitalizations were registered in 2014 in the SIH/SUS, of which 8,629,004 million (76.2%) were adults (20 or more years). Of these adult hospitalizations, 284,675 received an authorization for prolonged stay (AIH-5).

In 2014, an estimated 313,273 hospitalizations due to diabetes occurred in Brazil, corresponding to 3.6% of total hospitalizations and representing a hospitalization rate of 22.8/10,000 adults. Excluding hospitalizations for pregnancy, childbirth and the puerperium, hospitalizations attributable to diabetes represent 4.6% of total adult hospitalization in Brazil in 2014.

Among these, DM per se (ICD-10 codes E10–E14) accounted for 41.9% of hospitalizations, followed by cardiovascular diseases attributable to diabetes (26.5%) (Table 1). The population hospitalization rates increased from 3.5 and 3.8/10,000 adults for men and women, respectively, aged 20–44 years to 146.0 and 133.3 for the age group of 75 and over. Women were hospitalized more than men when considering both absolute number and crude hospitalization rate. However, when considering age-standardized rates, these are higher for men (23.9/10,000 population) when compared to women (21.9/10,000 population). While the average cost of a hospitalization of an adult individual was Int$709 in 2014, the average cost of a hospitalization due to diabetes and related diseases was 19% higher, reaching Int$845. Among the hospitalizations due to diabetes, hospitalizations due to kidney (Int$1602) and cardiovascular (Int$1529) diseases were the ones with higher average cost, and hospitalizations due to diabetes had the lower average cost (Int$364). Average hospitalization cost was significantly higher in men in all age groups and for all diagnosis groups, except for selected neoplasms, probably because of breast cancer costs included in this group (Table 2).

Total costs for adult hospitalization in the SUS in 2014 were approximately Int$6.1 billion. Admissions due to diabetes and related conditions reached Int$264.9 million, representing 4.3% of total hospitalization costs. After excluding hospitalizations for pregnancy, childbirth and the puerperium, this proportion increased to 4.8%. Diabetes mellitus per se accounted for only 18.1% of total costs attributable to hospitalization due to diabetes and related conditions, with cardiovascular diseases attributable to diabetes (47.9%) accounting for the higher proportion of overall costs. Total hospitalization costs were significantly higher in men from 20–74 years. The reverse was observed in the age group of 75 years and older (Table 3).

Among the hospitalizations with the main diagnosis reported as DM, the number of hospitalizations (52.5%), and total costs (46.2%) related to unspecified DM ICD-10 code E14) were the most observed, despite presenting the lower average hospitalization cost. The second most relevant cause of hospitalization in this group was insulin-dependent hospitalizations (ICD-10 E10), with the higher average hospitalization cost (Table 4).

Cardiovascular diseases due to diabetes accounted for 13.1% (n = 82,958) of admissions and 14.3% (Int$126,849,817) of costs of all hospitalizations for cardiovascular diseases in SUS. In hospitalizations due to diabetes, average hospitalization costs due to cardiovascular disease were 10.4% higher than non-diabetes hospitalizations. Among all hospitalizations due to cerebral infarction (ICD-10 code I63) and transient ischemic stroke and related syndromes (ICD-10 code G45), 25% of hospitalizations and costs could be attributed to diabetes (Table 5).

Microvascular diseases due to diabetes (kidney, eye and neurologic diseases) accounted for a greater share of total hospitalizations (29.1%) and associated costs (24.5%). Of worth noting is the high number of hospitalization and overall costs with diabetes hospitalization due to renal diseases, in particular due to chronic kidney disease (ICD-10 code N18) (Table 6).

Hospitalizations for respiratory and urinary infections, for which diabetes was considered a risk factor, represent a small percentage (5.3%) when compared to the cardiovascular (13.1%) and microvascular (29.1%) groups in relation to total SUS and accounted for 6.5% of hospitalizations due to DM. Even so, this percentage was reached due to the large participation of respiratory infections (96.5%) in this group, with emphasis on pneumonia per unspecified organism (ICD-10 J18) (69.9%) (Table 7).

Hospitalizations for neoplastic diseases have a small participation (7.3%) of total hospitalizations in comparison with other groups and represent 2.7% of hospitalizations due to DM. Breast cancer (4.0%) and colorectal cancer (5.8%) admissions were among those with the lowest values, while cancers of endometrium (20.0%), pancreas (18.6%) and liver and intrahepatic bile ducts (22.6%) were among those with the highest values (Table 8). 

## 4. Discussion

Brazil is one of the most populated countries in the world, with an estimated population of 137.6 million adults in 2014 [31]. Based on recent prevalence estimates, we have estimated that 17.3 million individuals aged 20 years and older had diabetes in Brazil (Supplementary Table S2). Despite increasing trends in diabetes prevalence in the country, mortality due to diabetes declined 1.7% per year (from 40.6/100 thousand population to 33.7/100 thousand population) from 2000 to 2011, probably as a result of better access to healthcare, thus reducing mortality due to acute events [32]. However, when diabetes was analyzed as an associated cause of death due to other causes, there was an increase of 8% between 2000 and 2007 [33], most likely representing deaths due to chronic diabetes complications and related conditions. 

Hospitalizations represent an important part of the consumption of health resources in different health systems and countries around the world and patients with type 2 diabetes had higher rates of hospitalization than the general population [34]. In the United States in 2012, diabetes hospitalization costs were the most significant cost component (43%) of direct medical costs ($176 billion) associated with diabetes, which added to $245 billion when considering both direct and indirect costs [25].

The estimated costs of hospitalizations due to diabetes and related conditions estimated in this study (Int$264.9 million) represent 4.6% of all hospitalizations and 0.45% of all expenditures for actions and public services of health provided by the Ministry of Health in 2014 (Int$58.3 billion) [35]. In this same year, total health expenditures in Brazil were 8% of its Gross Domestic Product of which 46% was associated with public health expenditures (Int$606 per capita) [36]. This spending is equivalent to Int$1.92 per adult by the federal government only with hospitalizations for DM and its complications. The average value of an adult hospitalization due to diabetes was 19% higher than a hospitalization without diabetes, and hospitalizations due to kidney and cardiovascular diseases were the ones with higher average cost.

Most countries in Latin America have adopted public health systems with universal coverage in the last few decades. Nonetheless, disparities in per capita government health expenditure can be observed in the region [37] and a wide difference can be identified between countries that share historic similarities, with Venezuela with the lowest (Int$270.88), and Cuba (Int$2366.06) the highest per capita government health expenditure [36]. When contrasting with high-income developed countries in other regions, disparities are more pronounced, with United States (Int$4541.17), United Kingdom (Int$2807.62), and Japan (Int$3115.08) among the highest per capita expenditures [36].

Our results demonstrated that the population aged 65 years and older used a much larger portion of hospital resources, both in number of hospitalization and costs, similar to results demonstrated in the United States in 2012 [25]. Cardiovascular complications attributable to diabetes also represented the largest share of all hospitalizations, both in number of hospitalizations and costs. 

Although when considering crude rates, women were more likely to be hospitalized than men, when adjusting for age taking into consideration the different age structure between men and women, men are more likely to be hospitalized than women. Although men had relatively higher hospitalization costs than women from age group 20–74 years, except for the ≥75 years age group, this may be due to the relatively longer life expectancy in women, compared with men [38]. Hospitalizations reported as having diabetes as the main diagnosis were the most frequent (41.9%), although with lower costs. They currently represent a small proportion of all hospitalization expenses for the Brazilian National Health System, but are expected to increase considerably as the population ages. Moreover, hospitalization costs related to diabetes, but not captured by a first listed diabetes diagnosis, must be integrated with these costs to give a more comprehensive picture of the overall disease burden attributable to diabetes.

The total number of hospitalizations due to DM-related conditions was 2.4 times that of hospitalizations for first-listed DM; however, spending was almost 5.5 times higher. Microvascular diseases due to diabetes (kidney, eye and neurologic diseases) accounted for a greater share of total hospitalizations (29.1%) and associated costs (24.5%), part of which could be prevented with a better metabolic control. These results were in accordance with others that show hospitalizations for diabetes complications had a higher average cost than those for diabetes itself [25,39,40].

The hospitalization costs due to infectious diseases and selected neoplasms in adults with DM were 4.8% and 3.9%, respectively, of the total hospitalizations due to DM. Although it represents a small percentage compared with vascular diseases, currently, this was the first Brazilian study to consider DM as an important contributor to such hospitalizations and costs.

Comparisons with Brazilian studies for 1999–2001 [41] and 2008–2010 [42], that used the same attributable risk methodology to estimate hospitalizations for DM in the Brazilian public network, should be performed with caution. In both previous studies, the results encompassed all age groups while in the current, only adults. In addition, in the two previous studies, hospitalizations were also estimated for the general medical conditions group, i.e., all other ICD-10 diseases that are not attributed to diabetes or its complications but for which individuals with DM were hospitalized more frequently. In contrast, in the current study, some of these conditions, such as certain neoplasms and lower respiratory and urinary tract infections, were computed as diabetes complications. It is also recognizable that the more recent literature has brought lower relative risks from international studies for the calculation of etiological fractions, although it was partially offset by the double of self-reported prevalence.

Our study has several limitations which are worth noting. The source of the data (SIH/SUS) was initially developed for administrative-financial functions for the purpose of collection and may not be free of coding errors, intentional or not. This is reflected by the high number of individuals hospitalized for whom the main diagnosis reported as DM was “unspecified DM—E14” (n = 68,987), and “insulin-dependent hospitalizations—E10” (n = 38,883), significantly higher than those reported as “non-insulin dependent diabetes—E11” (12,707). We believe that most of these cases reported as E14 are indeed individuals with type 2 DM. Also, many of the cases reported as E10 may be individuals with type 2 diabetes using insulin. 

In addition, the SUS covers 75% of the population in Brazil, which means about one quarter of population with diabetes was not included in the data analysis, and thus our estimates may be underestimated. Moreover, the diabetes hospitalization rates may be different among those not covered by SUS. As SIH data do not incorporate critical variables with explanatory potential, such as body mass index, race, schooling, severity of the clinical condition at the time of hospitalization, degree of use of the services, readmissions and other, we are not able to identify the role of these possible factors in diabetes hospitalizations.

Another limitation is we considered only the adult population, as the focus of the study was on type 2 DM, which is more amenable to prevention strategies. As such, although hospitalizations due to type 1 DM may have been inadvertently included in our estimation, on the other hand, we might have underestimated cases of type 2 DM in those younger than 20 years of age. 

Finally, as the more recent prevalence estimate available was that for self-reported diabetes [5], to account for undiagnosed diabetes, and as done by other authors [21], we applied a factor of 2, considering high quality recent evidence from Brazil [7]. The resulting diabetes prevalence of 12.4% considered in our analyses is consistent with sub-national studies in Brazil which a decade ago showed 2 digit prevalence figures in selected regions of the country, being 12.1% in the city of Ribeirão Preto [43], 12.4% in Porto Alegre [44], and 13.5% in São Carlos [45]. It is also consistent with prevalence estimates considered for global disease burden estimate studies [21].

## 5. Conclusions

This study presented a detailed overview of the hospitalizations attributable to DM in the Brazilian public network. It is a study that deals with the epidemiological and economic aspects of the public expenses with a disease. They portrayed an “epidemiological iceberg” present in developing societies such as Brazil. By increasing the incidence and severity of other diseases, diabetes increases the chances of hospitalization of patients and the use of more aggressive therapies. We believe that improving the quality of life of these patients depends, among other measures, on the expansion of preventive activities in order to reduce the need for hospitalization, minimize complications and reduce the severity of other more general medical conditions. Our estimate is part of the monitoring and analysis of the health situation for the necessary interventions. Understanding the costs of diabetes and its major complications is crucial to raise awareness and allow the assessment of strategies to reduce its prevalence and their impact.

## Figures and Tables

**Table 1:** Number and rates of hospitalization due to diabetes and related conditions, by age-group and sex, Unified Health System (SUS), Brazil, 2014.

	Age Groups (Years)										
Diabetes and Related Conditions	20–44		45–64		65–74		75+		Total		
	(n)		(n)		(n)		(n)		(n)		
	Men	Women	Men	Women	Men	Women	Men	Women	Men	Women	All
Diabetes Mellitus	8898	10,243	25,991	27,141	14,014	17,689	10,465	16,931	59,368	72,004	131,372
Attributed to diabetes											
Cardiovascular Disease *	1588	1683	14,527	12,046	14,482	13,536	10,683	14,412	41,281	41,678	82,958
Kidney Disease	1017	1170	4066	3385	3750	2844	3475	2852	12,308	10,251	22,559
Eye Disease	315	216	2417	2885	3400	5326	2333	3922	8465	12,349	20,814
Neurological Disease **	1668	976	6200	4561	4165	3268	2820	3050	14,853	11,855	26,708
Infectious Disease ***	564	685	1847	1866	2547	2614	4405	5846	9362	11,011	20,373
Neoplasms ****	104	439	1105	2332	1016	1865	542	1085	2767	5721	8488
Total *****	14,154	15,412	56,154	54,217	43,374	47,142	34,723	48,098	148,404	164,869	313,273
Crude Rate/10,000 population	3.5	3.8	28.4	25.4	101.6	90.1	146.0	133.3	22.2	23.3	22.8
Age adjusted Rate/10,000 population									23.9	21.9	

* Coronary heart disease and cerebrovascular disease; ** Diagnoses related to diabetic neuropathy; *** Urinary and respiratory infections; **** Breast, endometrial, pancreas, colorectal, hepatocarcinoma, cholangiocarcinoma. ***** Numbers do not necessarily sum to totals because of rounding.

**Table 2:** Average hospitalization cost (Int$) due to diabetes and related conditions by age-group and sex, SUS, Brazil, 2014.

	Age Groups (Years)										
Diabetes and Related Conditions	20–44		45–64		65–74		75+		Total		
									(Int$)		
	Men	Women	Men	Women	Men	Women	Men	Women	Men	Women	All
Diabetes Mellitus	551	516	355	319	360	321	340	324	383	349	364
Attributed to diabetes											
Cardiovascular Disease *	1245	916	1960	1469	1908	1478	1364	1036	1760	1300	1529
Kidney Disease	3393	2543	2292	1909	1400	1189	823	853	1696	1488	1602
Eye Disease	1324	1206	917	750	627	525	497	455	700	567	621
Neurological Disease **	545	486	653	595	745	691	807	769	696	657	679
Infectious Disease ***	578	435	669	607	681	635	615	620	642	610	624
Neoplasms ****	1054	1146	1135	1214	1250	1230	1233	1214	1194	1214	1207
Total *****	855	736	994	768	1065	808	801	664	956	746	845

* Coronary heart disease and cerebrovascular disease; ** Diagnoses related to diabetic neuropathy; *** Urinary and respiratory infections; **** Breast, endometrial, pancreas, colorectal, hepatocarcinoma, cholangiocarcinoma. ***** Numbers do not necessarily sum to totals because of rounding.

**Table 3:** Total hospitalization cost (in 000 Int$) due to diabetes and related conditions by age group and sex, SUS, Brazil, 2014.

	Age Groups (Years)										
Diabetes and Related Conditions	20–44		45–64		65–74		75+		Total		
	Men	Women	Men	Women	Men	Women	Men	Women	Men	Women	All
Diabetes Mellitus	4906.8	5287.7	9231.7	8650.5	5050.5	5684.6	3559.1	5489.5	22,747.9	25,112.4	47,860.3
Attributed to diabetes											
Cardiovascular Disease *	1977.4	1540.9	28,480.1	17,698.6	27,636.5	20,006.1	14,573.2	14,934.9	72,667.3	54,180.5	126,847.8
Kidney Disease	3449.4	2975.7	9320.8	6461.9	5250.6	3380.8	2859.6	2432.4	20,880.3	15,250.9	36,131.2
Eye Disease	416.9	260.7	2217.3	2162.8	2133.3	2798.2	1159.3	1782.6	5926.8	7004.3	12,931.1
Neurological Disease **	909.6	475.0	4049.4	2713.4	3101.1	2258.1	2276.1	2346.6	10,336.2	7793.1	18,129.3
Infectious Disease ***	325.6	298.0	1236.4	1132.6	1734.8	1660.2	2709.9	3624.1	6006.7	6714.9	12,721.6
Neoplasms ****	109.7	502.4	1254.6	2831.1	1270.1	2292.8	668.2	1317.9	3302.4	6944.2	10,246.6
Total *****	12,095.4	11,340.6	55,790.3	41,650.9	46,176.9	38,080.8	27,805.4	31,928	141,867.8	123,000.3	264,867.9

* Coronary heart disease and cerebrovascular disease; ** Diagnoses related to diabetic neuropathy; *** Urinary and respiratory infections; **** Breast, endometrial, pancreas, colorectal, hepatocarcinoma, cholangiocarcinoma. ***** Numbers do not necessarily sum to totals because of rounding.

**Table 4:** Number, average and total hospitalization cost due to diabetes (E10–E14), adults (20+ years), SUS, Brazil, 2014.

Diabetes Codes	Number	Average Hospitalization Cost	Total Hospitalization Cost
	(n)	(Int$)	(in 000 Int$)
E10 Insulin-dependent diabetes mellitus	38,883	452.62	17,599.3
E11 Non-insulin-dependent diabetes mellitus	12,707	340.82	4330.7
E12 Malnutrition-related diabetes mellitus	1454	384.83	559.5
E13 Other specified diabetes mellitus	9341	349.68	3266.4
E14 Unspecified diabetes mellitus	68,987	320.41	22,104.3
Total Diabetes (E10–E14) *	131,372	364.31	47,860.2

* Numbers do not necessarily sum to totals because of rounding.

**Table 5:** Number, average and total hospitalization cost due to cardiovascular disease, overall and related to diabetes, adults (20+ years), SUS, Brazil, 2014.

	Overall Hospitalization			Hospitalization Due to Diabetes		
Diabetes and Related Conditions	Number	Average Hospitalization Cost	Total Hospitalization Cost	Number	Average Hospitalization Cost	Total Hospitalization Cost
	(n)	(Int$)	(in 000 Int$)	(n)	(Int$)	(in 000 Int$)
I20 Angina pectoris	123,897	2265.29	280,662.5	21,202	2318.49	49,156.1
I21 Acute myocardial infarction	91,951	2025.70	186,265.3	13,784	2036.35	28,070.1
I23 Certain current complications following acute myocardial infarction	937	2069.76	1939.4	137	2293.55	315.1
I24 Other acute ischemic heart diseases	19,283	2878.38	55,503.8	3005	3009.33	9041.7
I22 Subsequent myocardial infarction	2248	1669.43	3752.9	340	1720.45	584.4
I25 Chronic ischemic heart disease	14,856	4065.16	60,392.1	2712	4065.62	11,027.9
I10 Essential (primary) hypertension	74,141	202.77	15,033.7	10,075	228.94	2306.6
I11 Hypertensive heart disease	9704	244.19	2369.6	736	271.00	199.5
I12 Hypertensive renal disease	1159	1736.11	2012.2	254	1277.07	324.6
I50 Heart failure	220,476	790.71	174,333.6	19,892	776.83	15,453
I60 Subarachnoid haemorrhage	9406	3339.63	31,412.6	259	3129.97	810.4
I61 Intracerebral haemorrhage	13,031	1555.16	20,265.3	404	1507.63	608.6
I62 Other non-traumatic intracranial haemorrhage	3736	2118.63	7915.2	113	2033.32	229.3
I63 Cerebral infarction	15,523	909.46	14,117.5	3787	920.99	3487.8
I65 Occlusion and stenosis of precerebral arteries, not resulting in cerebral infarction	2775	2782.18	7720.5	115	2755.04	315.5
I66 Occlusion and stenosis of cerebral arteries, not resulting in cerebral infarction	1255	840.22	1054.5	45	784.87	35.2
I67.2 Cerebral atherosclerosis	49	1303.74	63.9	10	1313.46	12.9
I69 Sequelae of cerebrovascular disease	7642	1577.12	12,052.4	1096	1837.99	2014.7
G45 Transient cerebral ischemic attacks and related syndromes	20,969	573.59	12,027.6	4993	571.77	2854.6
Total cardiovascular disease *	633,038	1404.17	888,894.5	82,958	1529.05	126,847.9

* Numbers do not necessarily sum to totals because of rounding.

**Table 6:** Number, average and total hospitalization cost due to renal, ophthalmological and neurological diseases, overall and related to diabetes microvascular complications, adults (20+ years), SUS, Brazil, 2014.

	Overall Hospitalization			Hospitalization Due to Diabetes		
Diabetes and Related Conditions	Number	Average Hospitalization Cost	Total Hospitalization Cost	Number	Average Hospitalization Cost	Total Hospitalization Cost
	(n)	(Int$)	(in 000 Int$)	(n)	(Int$)	(in 000 Int$)
Renal diseases						
N04 Nephrotic syndrome	2156	362.89	782.4	313	379.81	119.1
R77.0 Abnormality of albumin	-	-	-	-	-	-
R80 Isolated proteinuria	2	72.08	144	0.2	74.58	15
N17 Acute renal failure	21,960	1058.82	23,251.7	5555	1030.23	5722.6
N18 Chronic kidney disease	71,720	2243.99	160,939.2	16,678	1816.06	30,288.3
N19 Unspecified kidney failure	48	162.37	7.8	13	102.91	1.3
*Sub-total renal disease*	95,886	1929.18	184,981.3	22,559	1601.63	36,131.2
Eye diseases						
H25 Senile cataract	37,852	343.59	13,005.7	15,947	342.19	5456.9
H28 Cataract and other disorders of lens in diseases classified elsewhere	46	289.47	13.3	17	277.92	4.6
H33 Retinal detachments and breaks	15,858	1560.10	24,740.1	4773	1560.32	7447.01
H34 Retinal vascular occlusions	8	55.06	440	3	52.36	143
H35.0 Background retinopathy and retinal vascular changes	5	105.61	528	2	41.76	80
H35.2 Other proliferative retinopathy	-	-	-	-	-	-
H36.0 Retinal disorders in diseases classified elsewhere	159	128.45	20.4	53	129.84	6.9
H42 Glaucoma in diseases classified elsewhere	21	305.80	6.4	6	313.53	1.8
H54 Visual impairment including blindness (binocular or monocular)	56	837.97	46.9	14	968.52	13.5
*Sub-total ophthalmological disease*	54,005	700.56	37,833.9	20,814	621.26	12,931.1
Neurological diseases						
G90 Disorders of autonomic nervous system	205	1347.22	276.9	23	1821.26	41.5
G56 Mononeuropathies of upper limb	13,303	251.55	3346.3	1678	250.63	420.5
G57 Mononeuropathies of lower limb	223	522.86	116.6	24	585.04	14,3
G59.0 Diabetic mononeuropathy	5	163.55	818	1	174.99	169
G63 Polyneuropathy in diseases classified elsewhere	2407	355.05	854.6	334	347.54	116.1
G52 Disorders of other cranial nerves	239	1319.55	315.4	30	1253.71	37.4
L97 Ulcer of lower limb, not elsewhere classified	30,145	527.87	15,912.6	4673	534.64	2498.4
S88 Traumatic amputation of lower leg	1031	1040.48	1072.7	638	1023.73	653.1
S98 Traumatic amputation of ankle and foot	2586	397.03	1026.7	984	455.94	448.8
R02 Gangrene, not elsewhere classified	25,564	678.54	17,346.1	13,107	730.75	9577.8
M86 Osteomyelitis	13,209	572.71	7564.9	4480	625.41	2801.8
M87 Osteonecrosis	1785	1859.59	3319.4	736	2064.04	1520.01
*Sub-total neurological disease*	90,702	563.96	51,152.3	26,708	678.80	18,129.3
Total renal, ophthalmological and neurological disease *	240,593	1138.72	273,967.4	70,081	958.77	67,191.6

* Numbers do not necessarily sum to totals because of rounding.

**Table 7:** Number, average and total hospitalization cost due to respiratory and urinary infectious diseases, overall and related to diabetes, adults (20+ years), SUS, Brazil 2014.

	Overall Hospitalization			Hospitalization Due to Diabetes		
Diabetes and Related Conditions	Number	Average Hospitalization Cost	Total Hospitalization Cost	Number	Average Hospitalization Cost	Total Hospitalization Cost
	(n)	(Int$)	(in 000 Int$)	(n)	(Int$)	(in 000 Int$)
Respiratory infections						
J12 Viral pneumonia, not elsewhere classified	22,596	499.97	11,297.3	1075	530.04	569.9
J13 Pneumonia due to Streptococcus pneumoniae	1315	508.58	668.8	76	520.54	39.6
J14 Pneumonia due to Haemophilus influenzae	288	435.05	125.3	13	422.61	5.7
J15 Bacterial pneumonia, not elsewhere classified	79,130	679.63	53,778.8	4250	712.67	3028.8
J18 Pneumonia, organism unspecified	254,891	611.59	155,889.9	14,246	624.25	8893.1
*Sub-total lower respiratory tract infections*	358,220	619.06	221,760.1	19,661	637.67	12,537.1
Urinary tract infections						
N10 Acute tubulo-interstitial nephritis	20,247	200.30	4055.5	472	232.49	109.7
N15.1 Renal and perinephric abscess	699	1040.41	727.2	17	1071.13	18.1
N30.0 Acute cystitis	6873	217.55	1495.2	180	257.28	46.3
N30.8 Other cystitis	1718	215.64	370.5	44	237.58	10.4
*Sub-total urinary tract infections*	29,537	225.09	6648.5	712	258.93	184.5
Total infectious disease *	387,757	589.05	228,408.6	20,373	624.43	12,721.6

* Numbers do not necessarily sum to totals because of rounding.

**Table 8:** Number, average and total hospitalization cost due to neoplasms, overall and related to diabetes, adults (20+ years), SUS, Brazil, 2014.

	Overall Hospitalization			Hospitalization Due to Diabetes		
Diabetes and Related Conditions	Number	Average Hospitalization Cost	Total Hospitalization Cost	Number	Average Hospitalization Cost	Total Hospitalization Cost
	(n)	(Int$)	(in 000 Int$)	(n)	(Int$)	(in 000 Int$)
Breast						
C50 Malignant neoplasm of breast	55,580	1160.08	64,477.2	2200	1146.46	2522.2
D05.9 Carcinoma in situ of breast, unspecified	1192	989.73	1179.8	44	999.72	43.5
*Sub-total breast cancer*	56,772	1156.50	65,656.9	2244	1143.61	2565.7
Endometrium						
C54.1 Malignant neoplasm of corpus uteri	3539	1409.76	4989.1	716	1429.85	1023.4
D07.0 Carcinoma in situ of other and unspecified genital organs	202	151.49	30.6	31	158.36	4.9
*Sub-total endometrium cancer*	3741	1341.82	5019.7	747	1376.34	1028.4
Pancreas						
C25 Malignant neoplasm of pancreas	7867	1173.41	9231.2	1464	1128.19	1652.2
*Sub-total pancreas cancer*	7867	1173.41	9231.2	1464	1128.19	1652.2
Liver and intrahepatic bile ducts						
C22.1 Intrahepatic bile duct carcinoma	807	1082.93	873.9	151	1069.35	161.9
C22.0 Liver cell carcinoma	2517	1306.91	3289.5	589	1282.92	755.4
C22.7 Other specified carcinomas of liver	882	1545.40	1363.0	197	1455.41	287.1
C22.9 Malignant neoplasm of liver and intrahepatic bile ducts—liver, unspecified	3158	547.64	1729.4	726	527.42	383.2
*Sub-total liver and cholangiocarcinoma cancer*	7364	985.32	7255.9	1664	954.10	1587.6
Colorectal						
C18 Malignant neoplasm of colon	37,627	1238.60	46,604.8	2187	1329.29	2907.7
C19 Malignant neoplasm of recto sigmoid junction	2946	2727.89	8036.4	181	2787.70	505.01
*Sub-total colorectal cancer*	40,573	1346.74	54,641.2	2369	1440.83	3412.7
Total neoplasms disease *	116,317	1219.13	141,804.9	8488	1207.23	10,246.6

* Numbers do not necessarily sum to totals because of rounding.

## Supplementary Materials

The following are available online at http://www.mdpi.com/1660-4601/15/2/294/s1, Table S1: DM and related conditions and relative risks, Table S2: State level prevalence and hospitalization cost due to diabetes and related conditions, adults (20+ years), SUS, Brazil, 2014.
