# Supplementary material for: Disease and Economic Burden of Hospitalizations Attributable to Diabetes Mellitus and Its Complications: A Nationwide Study in Brazil
Source: Int J Environ Res Public Health. 2018 Feb 8;15(2):294. doi: 10.3390/ijerph15020294 (PMC5858363; doi:10.3390/ijerph15020294)
Supplement: Supplementary file 1 [file ijerph-15-00294-s001.pdf]

International Journal of
Environmental Research and Public Health

MDPI

Table S1. DM and related conditions and relative risks.

	Men	Women	Both	Reference
**Cardiovascular Disease**				
I20 Angina pectoris			1.89	[3]
I21 Acute myocardial infarction			1.74	[3]
I23 Certain current complications following acute myocardial infarction			1.74	[3]
I24 Other acute ischaemic heart diseases			1.74	[3]
I22 Subsequent myocardial infarction			1.74	[3]
I25 Chronic ischaemic heart disease			1.89	[3]
I10 Essential (primary) hypertension			1.68	[1]
I11 Hypertensive heart disease			1.36	[4]
I12 Hypertensive renal disease			2.55	[2]
I50 Heart failure			1.36	[4]
I60 Subarachnoid haemorrhage			1.14	[3]
I61 Intracerebral haemorrhage			1.14	[3]
I62 Other non-traumatic intracranial haemorrhage			1.14	[3]
I63 Cerebral infarction				[5]
Age group (years) 30–44			5.60	
45–59			3.60	
60–74			2.10	
>=75			1.80	
I65 Occlusion and stenosis of precerebral arteries, not resulting in cerebral infarction			1.14	[3]
I66 Occlusion and stenosis of cerebral arteries, not resulting in cerebral infarction			1.14	[3]
I67.2 Cerebral atherosclerosis			1.76	[3]
I69 Sequelae of cerebrovascular disease			1.76	[3]
G45 Transient cerebral ischemic attacks and related syndromes				[5]
Age group (years) 30–44			5.60	
45–59			3.60	
60–74			2.10	

75+	1.80	
**Renal diseases**	2.55	[2]
N04 Nephrotic syndrome		
R77.0 Abnormality of albumin		
R80 Isolated proteinuria		
N17 Acute renal failure		
N18 Chronic kidney disease		
N19 Unspecified kidney failure		
**Eye diseases**	3.22	[2]
H25 Senile cataract		
H28 Cataract and other disorders of lens in diseases classified elsewhere		
H33 Retinal detachments and breaks		
H34 Retinal vascular occlusions		
H35.0 Background retinopathy and retinal vascular changes		
H35.2 Other proliferative retinopathy		
H36.0 Retinal disorders in diseases classified elsewhere		
H42 Glaucoma in diseases classified elsewhere		
H54 Visual impairment including blindness (binocular or monocular)		
**Neurological diseases**		
G90 Disorders of autonomic nervous system	1.97	[2]
G56 Mononeuropathies of upper limb	1.97	[2]
G57 Mononeuropathies of lower limb	1.97	[2]
G59.0 Diabetic mononeuropathy	1.97	[2]
G63 Polyneuropathy in diseases classified elsewhere	1.97	[2]
G52 Disorders of other cranial nerves	1.97	[2]
L97 Ulcer of lower limb, not elsewhere classified	1.97	[2]
S88 Traumatic amputation of lower leg		[6]
Age group (years) 35–44	3.04	
45–54	9.82	
55–59	22.53	
60–64	35.36	

65–74			63.52	
75+			163.49	
S98 Traumatic amputation of ankle and foot				[6]
Age group (years) 35–44			1.75	
45–54			5.01	
55–59			7.68	
60–64			9.86	
65–74			12.15	
75+			15.77	
R02 Gangrene, not elsewhere classified				[6]
Age group (years) 35–44			2.07	
45–54			6.50	
55–59			11.81	
60–64			16.49	
65–74			23.88	
75+			43.92	
M86 Osteomyelitis			5.80	[7]
M87 Osteonecrosis			5.80	[7]
**Respiratory and urinary infectious diseases**				
N10 Acute tubulo-interstitial nephritis				[8]
Age group (years) 18–24	1.17	1.19		
25–34	1.21	1.19		
35–44	1.21	1.18		
45–54	1.21	1.13		
55–64	1.19	1.13		
65–74	1.16	1.14		
75+	1.16	1.12		
N15.1 Renal and perinephric abscess				[8]
Age group (years) 18–24	1.17	1.19		
25–34	1.21	1.19		
35–44	1.21	1.18		

45–54	1.21	1.13		
55–64	1.19	1.13		
65–74	1.16	1.14		
75+	1.16	1.12		
N30.0 Acute cystitis				[8]
Age group (years) 18–24	1.17	1.19		
25–34	1.21	1.19		
35–44	1.21	1.18		
45–54	1.21	1.13		
55–64	1.19	1.13		
65–74	1.16	1.14		
75+	1.16	1.12		
N30.8 Other cystitis				[8]
Age group (years) 18–24	1.17	1.19		
25–34	1.21	1.19		
35–44	1.21	1.18		
45–54	1.21	1.13		
55–64	1.19	1.13		
65–74	1.16	1.14		
75+	1.16	1.12		
Respiratory infections			1.23	[9]
J12 Viral pneumonia, not elsewhere classified				
J13 Pneumonia due to Streptococcus pneumoniae				
J14 Pneumonia due to Haemophilusinfluenzae				
J15 Bacterial pneumonia, not elsewhere classified				
J18 Pneumonia, organism unspecified				
**Neoplasms**				
Breast			1.20	[10]
C50 Malignant neoplasm of breast				
D05.9 Carcinoma in situ of breast, unspecified				
Liver and intrahepatic bile ducts				

C22.1 Intrahepatic bile duct carcinoma	1.97	[10]
C22.0 Liver cell carcinoma	2.31	[11]
C22.7 Other specified carcinomas of liver	2.31	[11]
C22.9 Malignant neoplasm of liver and intrahepatic bile ducts—liver, unspecified	2.31	[11]
Colorectal	1.27	[10]
C18 Malignant neoplasm of colon		
C19 Malignant neoplasm of recto sigmoid junction		
Endometrium	1.97	[10]
C54.1 Malignant neoplasm of corpus uteri		
D07.0 Carcinoma in situ of other and unspecified genital organs		
Pancreas	1.94	[12]
C25 Malignant neoplasm of pancreas		

Notes: Ref. [1]—RR calculated based on self-report prevalence of hypertension for diabetics in relation to non-diabetics according to the National Health Survey (PNS) of 2013. Ref. [4]—RR calculated according Grant (2014) [13].

Table S2. State level prevalence and hospitalization cost due to diabetes and related conditions, adults (20+ years), SUS, Brazil, 2014.

State	Prevalence (%)	Population with Diabetes	Hospitalization (n)	Cost (in 000)
Rondônia	10.0	112,492	3511	1716.1
Acre	9.9	43,930	765	410.2
Amazonas	9.5	212,302	2862	1842.6
Roraima	9.0	25,459	745	376.5
Pará	10.3	495,189	9561	4068.2
Amapá	9.1	38,037	651	467.7
Tocantins	10.7	99,954	2859	1558.7
North region	*10.0*	*1,027,363*	*20,955*	*10,439.4*
Maranhão	11.1	449,367	11,976	4504.3
Piauí	12.1	247,729	6258	2358.8
Ceará	12.2	701,396	9070	7427.5
Rio Grande do Norte	12.2	276,781	4398	3574.6
Paraíba	12.8	333,033	4974	3178.1
Pernambuco	12.4	753,867	13,149	12,170.7
Alagoas	11.6	237,841	4554	3327.3
Sergipe	11.4	161,745	1745	1367.6
Bahia	12.1	1,211,736	27,944	14,003.8
Notheast region	*12.0*	*4,373,495*	*84,068*	*51,912.8*
Minas Gerais	13.1	1,895,874	38,797	35,628.5
Espírito Santo	12.3	329,834	5835	5207.4
Rio de Janeiro	14.2	1,669,845	18,093	16,846.7
São Paulo	13.1	4,102,950	66,884	70,818.1
Southeast region	*13.3*	*7,998,504*	*129,608*	*128,500.7*
Paraná	12.9	994,666	19,832	20,924.9
Santa Catarina	12.5	592,684	10,489	11,034.4
Rio Grande do Sul	14.5	1,169,597	25,247	24,056.9
South region	*13.4*	*2,756,947*	*55,568*	*56,016.3*
Mato Grosso do Sul	11.8	206,552	5116	4121.7
Mato Grosso	10.7	227,248	4806	2859.4
Goiás	11.5	509,315	10,080	8198.9

Distrito Federal	11.2	220,916	3071	2818.2
Midwest region	*11.3*	*1,164,030*	*23,074*	*17,998.2*
TOTAL *	12.6	17,320,339	313,273	264,867.9

Note: The prevalence and population of diabetics correspond to the sum of the estimates calculated specifically for each sex, age at 13 intervals every 5 years from 20 years old and state from the data of self-reported prevalence obtained accordingly from the National Health Survey 2013 multiplied by 2 (see methodological section). * Numbers do not necessarily sum to totals because of rounding.

References

1. Instituto Brasileiro de Geografia e Estatística (IBGE). *Pesquisa Nacional de Saúde—2013: Percepção do Estado de Saúde, Estilos de Vida e Doenças Crônicas: Brasil, Grandes Regiões e Unidades da Federação*; IBGE: Rio de Janeiro, Brazil, 2014.
2. Donnan, P.T.; Leese, G.P.; Morris, A.D. Hospitalizations for people with type 1 and type 2 diabetes compared with the nondiabetic population of tayside, scotland: A retrospective cohort study of resource use. *Diabetes Care* **2000**, *23*, 1774–1779.
3. Sarwar, N.; Gao, P.; Seshasai, S.R.; Gobin, R.; Kaptoge, S.; Di Angelantonio, E.; Ingelsson, E.; Lawlor, D.A.; Selvin, E.; Stampfer, M.; et al. Diabetes mellitus, fasting blood glucose concentration, and risk of vascular disease: A collaborative meta-analysis of 102 prospective studies. *Lancet* **2010**, *375*, 2215–2222.
4. Kamalesh, M.; Cleophas, T.J. Heart failure due to systolic dysfunction and mortality in diabetes: Pooled analysis of 39,505 subjects. *J. Card. Fail.* **2009**, *15*, 305–309.
5. Jeerakathil, T.; Johnson, J.A.; Simpson, S.H.; Majumdar, S.R. Short-term risk for stroke is doubled in persons with newly treated type 2 diabetes compared with persons without diabetes: A population-based cohort study. *Stroke* **2007**, *38*, 1739–1743.
6. Lombardo, F.L.; Maggini, M.; De Bellis, A.; Seghieri, G.; Anichini, R. Lower extremity amputations in persons with and without diabetes in italy: 2001–2010. *PLoS ONE* **2014**, *9*, e86405.
7. Al-Mayahi, M.; Cian, A.; Kressmann, B.; de Kalbermatten, B.; Rohner, P.; Egloff, M.; Jafaar, J.; Malacarne, S.; Miozzari, H.H.; Uckay, I. Associations of diabetes mellitus with orthopaedic infections. *Infect. Dis. (Lond.)* **2016**, *48*, 70–73.
8. Fu, A.Z.; Iglay, K.; Qiu, Y.; Engel, S.; Shankar, R.; Brodovicz, K. Risk characterization for urinary tract infections in subjects with newly diagnosed type 2 diabetes. *J. Diabetes Complicat.* **2014**, *28*, 805–810.
9. Kornum, J.B.; Thomsen, R.W.; Riis, A.; Lervang, H.H.; Schonheyder, H.C.; Sorensen, H.T. Diabetes, glycemic control, and risk of hospitalization with pneumonia: A population-based case-control study. *Diabetes Care* **2008**, *31*, 1541–1545.
10. Tsilidis, K.K.; Kasimis, J.C.; Lopez, D.S.; Ntzani, E.E.; Ioannidis, J.P. Type 2 diabetes and cancer: Umbrella review of meta-analyses of observational studies. *BMJ* **2015**, *350*, doi:10.1136/bmj.g7607.
11. Wang, C.; Wang, X.; Gong, G.; Ben, Q.; Qiu, W.; Chen, Y.; Li, G.; Wang, L. Increased risk of hepatocellular carcinoma in patients with diabetes mellitus: A systematic review and meta-analysis of cohort studies. *Int. J. Cancer* **2012**, *130*, 1639–1648.
12. Ben, Q.; Xu, M.; Ning, X.; Liu, J.; Hong, S.; Huang, W.; Zhang, H.; Li, Z. Diabetes mellitus and risk of pancreatic cancer: A meta-analysis of cohort studies. *Eur. J. Cancer* **2011**, *47*, 1928–1937.
13. Grant, R.L. Converting an odds ratio to a range of plausible relative risks for better communication of research findings. *BMJ* **2014**, *348*, doi:10.1136/bmj.f7450.
